# Synergistic Regulation of Oxygen Reduction Activity on Antimonene via Transition Metal–Nonmetal Dual-Atom Doping

**DOI:** 10.3390/nano16080465

**Published:** 2026-04-14

**Authors:** Yusong Weng, Xin Zhao, Wentao Liang, Ming Wang, Wei Deng, Xuefei Liu

**Affiliations:** 1 School of Physics and Electronic Science, Guizhou Normal University, Guiyang 550025, China; 231108100030@gznu.edu.cn (Y.W.);; 2 School of Integrated Circuit, Guizhou Normal University, Guiyang 550025, China; 3 Guizhou Yiyun New Materials Technology Co., Ltd., Guiyang 561113, China; 4 School of Big Data Statistics, Guizhou University of Finance and Economics, Guiyang 550025, China

**Keywords:** antimonene, dual-atom co-doping, oxygen reduction reaction (ORR), density functional theory (DFT), synergistic effect

## Abstract

Two-dimensional antimonene has recently emerged as a promising electrocatalytic platform; however, its oxygen reduction reaction (ORR) activity and modulation strategies remain largely unexplored. Herein, density functional theory (DFT) calculations are employed to systematically investigate ORR catalysis on antimonene co-doped with transition metal (TM) and nonmetal (C, P) dual atoms. The results reveal that Pd@C–Sb, Pt@C–Sb, and Pd@P–Sb exhibit remarkably enhanced ORR activity, delivering low overpotentials of 0.31 V, 0.32 V, and 0.38 V, respectively, significantly outperforming their single-atom-doped counterparts. Mechanistic analyses demonstrate that nonmetal dopants induce strong synergistic interactions with TM centers, leading to charge redistribution and effective regulation of the TM d-band center, which optimizes the adsorption energetics of key ORR intermediates. Notably, the number of d-electrons of TM atoms is identified as a reliable electronic descriptor governing intermediate binding strength and catalytic activity. Furthermore, ab initio molecular dynamics simulations confirm the excellent thermodynamic stability of the optimized dual-atom catalysts. This work elucidates the atomic-scale origin of synergistic enhancement in dual-atom-doped antimonene and provides a rational design strategy for high-performance ORR electrocatalysts based on two-dimensional main-group materials.

## 1. Introduction

Considerable efforts have been devoted to the development of high-performance ORR catalysts for proton exchange membrane fuel cells (PEMFCs) and metal–air batteries, aiming to enhance energy conversion efficiency, lower material costs, and promote the sustainable utilization of energy resources [1,2,3,4,5,6,7,8,9,10]. An in-depth understanding of the electrocatalytic mechanism of ORR can facilitate targeted catalyst design and optimization of cell structures, which are critical for increasing ORR efficiency, improving the performance of energy conversion and storage devices, and advancing clean energy technologies [11,12,13]. Since the discovery of graphene, the research and development of two-dimensional materials (2D) has led to the emergence of a variety of new materials, such as phosphorene [14,15,16], transition metal disulfides [17,18,19], MXenes [20,21,22], and so on. The advantages of these 2D materials in electrocatalysis are reflected in their high specific surface area, tunable electronic structure, excellent electrical conductivity, and surface chemical activity [23,24,25,26], which provide an effective way to design and develop efficient and stable ORR electrocatalysts.

Antimonene, a relatively recent addition to the realm of 2D materials, had its existence foreseen in 2015 and was successfully isolated and synthesized in the laboratory by 2016 [27,28]. Comprising a single layer of antimony atoms arranged in a hexagonal honeycomb pattern, antimonene adopts a quasi-van der Waals phase structure, imparting unique interlayer characteristics and granting relative independence in three-dimensional space [29,30]. Meanwhile, the hexagonal lattice structure serves as the cornerstone for its electronic band structure and exceptional conductive properties [27]. In terms of electronic attributes, antimonene displays remarkable topological insulator properties [31]. Its energy band structure renders it an insulator in the interior while demonstrating superior electronic conductivity at the edges, offering robust support for efficient electron transport [32,33,34,35]. In the realm of electrocatalysis, 2D antimonene materials also showcase outstanding electrochemical properties. Recently, Xiaohui Ren et al. achieved the hydrogen evolution reaction (HER) and oxygen evolution reaction (OER) of 2D antimonene through experimental studies, demonstrating its bifunctional electrocatalytic activity and structural robustness [36]. Shihai Cao et al. demonstrated that the electrocatalytic nitrogen fixation performance could be enhanced through defect regulation of 2D antimonene [37]. Furthermore, Mengya Yang et al. discovered that the incorporation of a group VIA atom as a dopant could enhance the HER catalytic activity of 2D antimonene [38]. Nevertheless, there remains insufficient knowledge about this crucial electrocatalytic material, particularly in the discovery and design of efficient ORR catalysts [39].

In the ongoing exploration of catalytic science, numerous strategies for improving electrocatalytic activity have emerged, including alloying [40], porous structure design [41], heterogeneous structure construction [42], non-homogeneous phase catalysis [43], etc. Among these strategies, single-atom catalysis stands out as a pivotal approach, elevating efficiency through precise manipulation of catalytically active sites at the individual atomic level, thereby significantly enhancing electrocatalytic performance [44,45]. Furthermore, nonmetallic (NM) modulation has garnered increasing attention. NM atoms can modulate the electronic structure and material properties of the active centers on the surface of TM atom-doped single-atom catalysts (SACs), thus increasing their intrinsic catalytic activity [46,47]. Hence, the primary focus of this study will be on NM-modulated TM atom-doped 2D antimonene materials, aiming to enhance the electrocatalytic performance for ORR.

Recently, Cui et al. [48] reported that MnM–NC (M = Ga, In, Sn) dual-single-atom catalysts exhibit efficient ORR activity. Inspired by such dual-atom strategies, we herein investigate the ORR catalysis on antimonene co-doped with transition metals (Cr, Mn, Fe, Co, Ni, Cu, Pd, Pt) and nonmetals (C, P), denoted as TM@C/P–Sb. The outcomes obtained through DFT calculations indicate that the Pd@C–Sb (η_ORR_ = 0.31 V), Pt@C–Sb (η_ORR_ = 0.32 V), and Pd@P–Sb (η_ORR_ = 0.38 V) systems exhibit favorable ORR overpotentials, respectively. With excellent structural stability, these catalysts exhibit promising potential for ORR. Building upon this foundation, we observed that the number of electrons in the d orbitals of TM atoms serves as a reliable descriptor for gauging the binding strength between the intermediate and the substrate.

## 2. Computational Methods and Models

The geometric structures and initial models of the TM@C/P–Sb systems were constructed using the Device Studio program. The interaction between ion cores and valence electrons was described using the projector-augmented wave (PAW) method [49]. All structural optimizations were performed using the DS-PAW software package (version 2023B). The exchange–correlation interactions were treated using the Perdew–Burke–Ernzerhof (PBE) functional within the generalized gradient approximation (GGA) [50]. To accurately account for long-range van der Waals interactions, the DFT-D3 method with Grimme correction was employed, which has been widely validated in previous studies [51].

Following structural relaxation, electronic structure calculations and catalytic activity analyses were carried out using the Vienna Ab initio Simulation Package (VASP) [52]. The PBE functional within the GGA framework was employed. Van der Waals interactions were further corrected using the DFT-D3 method. The PAW method was used to describe the ion–electron interaction. To consider solvation effects, the VASPsol model was employed during the calculations [53].

DFT+U calculations were also considered to improve the description of localized d-electrons. Previous studies have shown that the U value has a limited influence on the adsorption behavior of single atoms on substrates [54,55]. Therefore, we evaluated the influence of U on ORR catalytic activity using parameters reported in the literature (UPt = 2.4 eV, UPd = 3.3 eV) [56].

The plane-wave cutoff energy was set to 400 eV. The convergence criteria for energy and force were 1.0 × 10^−5^ eV and 2.0 × 10^−2^ eV Å^−1^, respectively. A 2 × 2 × 1 Monkhorst–Pack k-point grid was used for Brillouin zone sampling.

Ab initio molecular dynamics (AIMD) simulations were performed at 400 K for 8 ps with a time step of 1 fs using the Nosé–Hoover thermostat to evaluate the thermal stability of the doped systems [57].

The ORR mechanism was investigated using the computational hydrogen electrode (CHE) model. Under acidic conditions, the four-electron pathway can be described as follows:

(1) $$O_{2}(g)+*+H^{+}+e^{-}\to \;*OOH$$

(2) $$*\;OOH+H^{+}+e^{-}\to *\;O+H_{2}O(l)$$

(3) $$*\;O+H^{+}+e^{-}\to *\;OH$$

(4) $$*\;OH+H^{+}+e^{-}\to H_{2}O(l)+*$$

The two-electron pathway is given by

(5) $$O_{2}(g)+*+H^{+}+e^{-}\to *\;OOH$$

(6) $$*\;OOH+H^{+}+e^{-}\to *+H_{2}O_{2}$$

where * denotes the active site, and (g) and (l) represent gas and liquid phases, respectively.

The Gibbs free energy change (ΔG) for each elementary step was calculated as follows:

(7) $$\Delta G=\Delta E+\Delta E\_ZPE-T\Delta S+\Delta G_{\mathrm{pH}}+\Delta G_{U}$$

where ΔE, ΔE_ZPE_, and TΔS represent the electronic energy change, zero-point energy correction, and entropy contribution at T = 298.15 K, respectively. The pH correction term is defined as ΔG_pH_ = 2.303k_BT_ × pH, where k_B_ is the Boltzmann constant. Under acidic conditions, pH = 0. The electrode potential correction is given by ΔG_U_ = −neU, where n is the number of transferred electrons, e is the elementary charge, and U is the applied potential. Additional computational details are provided in the Supplementary Information (SI).

## 3. Results and Discussion

### 3.1. Structural Features, Stabilities, and Active Sites of the TM@C/P–Sb

**Figure 1:**
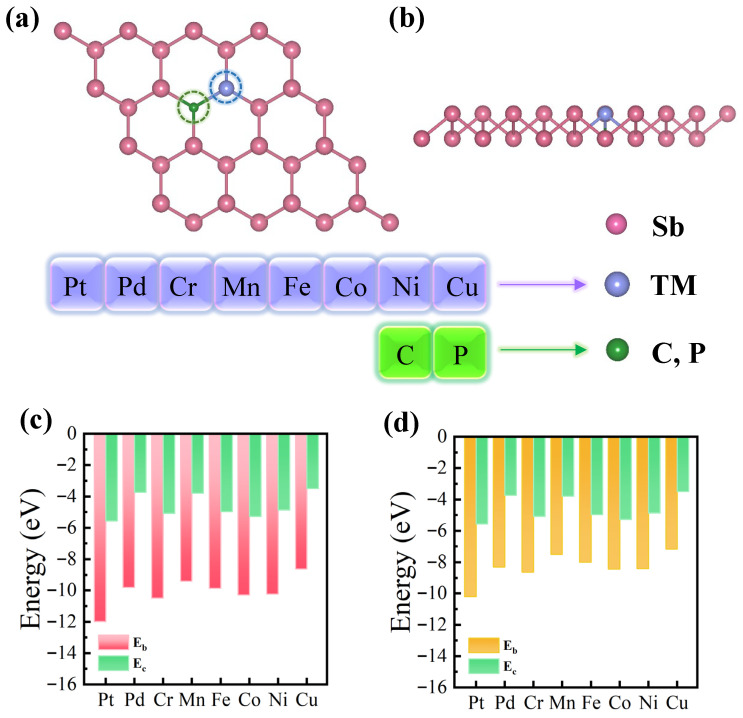
Top view (**a**) and side view (**b**) of a 4 × 4 × 1 supercell of Sb with a TM and an NM embedded within it, where pink, purple, and green spheres correspond to Sb, TM, and NM atoms, respectively; (**c**) corresponds to the binding energy (E_b_) of TM@C–Sb and the cohesive energy Ec (Equation (S2)) of the TM bulk; (**d**) corresponds to E_b_ and the cohesive energy Ec (Equation (S2)) of the TM bulk.

In this study, we initially created a 4 × 4 × 1 supercell of the Sb system. We then eliminated the atoms at two neighboring Sb sites within this supercell, generating two adjacent vacancies. Following this, a TM (TM = Pt, Pd, Cr, Mn, Fe, Co, Cu) and an NM (NM = C, P) atom were incorporated into these two vacancy structures, as illustrated in Figure 1a and Figure 1b, respectively. It is important to highlight that, to avoid disturbing the local crystal structure and increasing the total energy of the system when introducing atoms, we made the deliberate choice to introduce the TM and NM atoms after the formation of vacancies. This approach aims to decrease the system’s energy and enhance overall stability [58].

We define the vacancy formation energy of $V_{Sb}$ as $E_{f}(V_{Sb})=E_{V_{Sb}}-n\times E_{N}$, where $E_{V_{Sb}}$, n, and $E_{N}$ are the energies of the system with two neighboring Sb vacancies, as well as the number of Sb atoms in the original system and the corresponding energies, respectively, in order to study the structural stability of the TM@C/P–Sb systems. The calculated $E_{f}(V_{Sb})$ is 1.93 eV, which is smaller than that of graphene ($E_{f}(V_{C})=7.69\;eV$) [59], phosphorene ($E_{f}\left(V_{P}\right)=2.03\;eV$) [60], and MoS_2_ ($E_{f}\left(V_{S}\right)=5.85\;eV$) [61], among others. The magnitude of the vacancy formation energy directly influences the stability of the vacancies in the material and the difficulty in forming them. A lower vacancy formation energy indicates that less energy is required to form vacancies, and therefore, the Sb vacancy structure is easier to synthesize experimentally [58]. The difference between the binding and cohesion energies is commonly used to assess the stability and immobilization of the active site in a catalyst and can be interpreted as the relative stability of the active site and its surroundings with respect to the internal interbonding. A larger difference between these two energy parameters implies a more stable active site on the catalyst [62,63]. Table S1 summarizes the binding energy (E_b_, Equation (S1)) for all systems post-doping and the cohesion energy (E_c_, Equation (S2)) introduced into the transition metal block. From the calculated results, it is evident that *E_b_* is consistently negative across all systems, indicating structural stability from a thermodynamic perspective. When comparing these results with the calculations of E_c_, as illustrated in Figure 1c,d, it becomes apparent that the E_b_ values for all doped systems are considerably more negative than E_c_. This substantial negativity in E_b_ assists in restraining the mutual attraction between transition metals, thereby averting unnecessary structural changes and agglomeration. Consequently, this phenomenon contributes to enhancing the long-term catalytic performance of the catalysts.

### 3.2. ORR Activity

**Figure 2:**
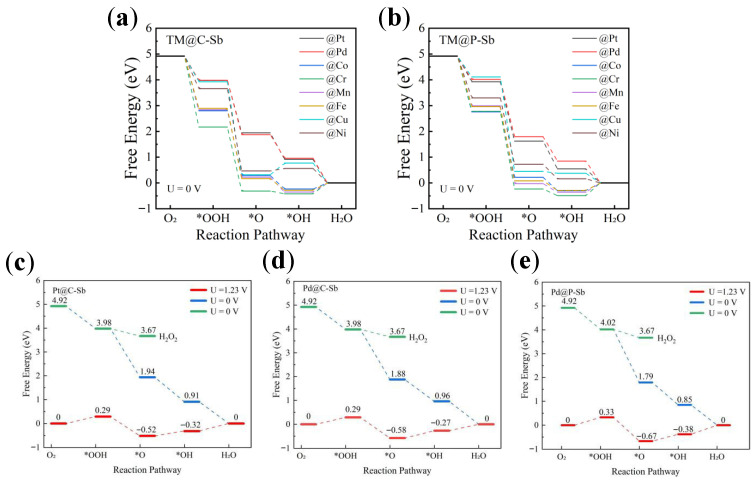
Free energy step diagrams for the ORR of TM@C–Sb (**a**) and TM@P–Sb (**b**); ΔG diagrams for the basic steps of the ORR of Pt@C–Sb (**c**), Pd@C–Sb (**d**), and Pd@P–Sb (**e**) at different potentials.

The overall process of ORR can be divided into four basic steps (Equations (1)–(4)), which are carried out sequentially at the active sites on the electrocatalyst surface. The ΔG differences (Equations (S3)–(S6)) of adsorbed intermediates ($\Delta G_{*OOH}$, $\Delta G_{*O}$ and $\Delta G_{*OH}$) are critical indicators for assessing the ORR. Thus, we fixed the starting positions of OOH, O, and OH radical intermediates on TM and investigated the free energy differences in the TM@C/P–Sb systems to provide information about the stability and reactivity of the reaction intermediates during catalysis. The Gibbs free energies of the intermediates and the Gibbs free energy differences between two adjacent reaction steps are summarized in Tables S2 and S3, respectively. The free energy step diagrams of the reaction process for the ORR of all TM@C/P–Sb systems are shown in Figure 2a,b. In order to more accurately model the kinetic and thermodynamic properties of the ORR, a standard electrode potential of 1.23 V for the reduction of oxygen molecules to water was introduced into the potential step diagrams for the three systems in which the η_ORR_ was relatively small (Figure 2c–e). The energy barrier of each step in the ORR process is directly related to the rate of the reaction, and the step with the highest potential barrier requires the greatest amount of energy to overcome; therefore, it is usually considered to be the decisive potential step of the whole ORR process. It can be concluded that, in the TM@C/P–Sb systems (Cu@C–Sb, Ni@C–Sb, Pd@C–Sb, and Cu@P–Sb), the third step of generating *OH in the ORR process consumes the largest amount of energy, with $\Delta G_{*OH}$ values of 0.56, 0.77, 0.96, and 0.16 eV, respectively, whereas the step with the largest amount of energy consumption for the rest of the systems is the fourth step of the reaction that generates H_2_O. In all systems, Pd@C–Sb, Pt@C–Sb, and Pd@P–Sb have relatively small η_ORR_ of 0.31 V, 0.32 V, and 0.38 V, respectively. Interestingly, based on previous research data [62,63], under equivalent conditions, compared to single TM atoms as electrocatalytic active sites doped on an Sb substrate (e.g., Pd@Sb = 0.58 V and Pt@Sb = 0.71 V), the η_ORR_ for the TM@C–Sb and TM@P–Sb systems exhibited an average reduction of 50.74% and 17.26%, respectively. It can be seen that our introduction of C or P atoms into the Sb substrate to modulate the TM active site can improve the electrocatalytic activity of ORR and can be an effective way to develop more efficient and stable electrocatalysts. In addition, to better capture the electronic correlation of transition metals, we also considered the effect of GGA + U on the ORR of Pd@C–Sb, Pt@C–Sb, and Pd@P–Sb systems, as shown in Table S4. According to the calculation results, the η_ORR_ value after + U exhibited a small change, and the correction range was 0.003~0.049 V.

To evaluate the selectivity of the catalysts toward the 4-electron ORR pathway, we further calculated the ΔG changes for the 2-electron pathway (O_2_ → *OOH → H_2_O_2_) under acidic conditions. As summarized in Figure 2c–e, the free energy barrier for the rate-determining step of the 2-electron pathway (*OOH → H_2_O_2_) is significantly higher than that of the 4-electron pathway for all three representative systems. For instance, in Pd@C–Sb, the ΔG for *OOH → H_2_O_2_ is 1.52 eV at U = 0 V, while the rate-determining step for the 4-electron pathway (*OH → H_2_O) has a ΔG of only 1.00 eV. Similar trends are observed for Pt@C–Sb (1.45 eV vs. 0.91 eV) and Pd@P–Sb (1.48 eV vs. 0.85 eV). These results indicate that the 4-electron pathway is thermodynamically more favorable, confirming that Pd@C–Sb, Pt@C–Sb, and Pd@P–Sb exhibit high selectivity toward the 4-electron ORR with H_2_O production.

### 3.3. Scaling Relations and Electronic Structure Analysis

**Figure 3:**
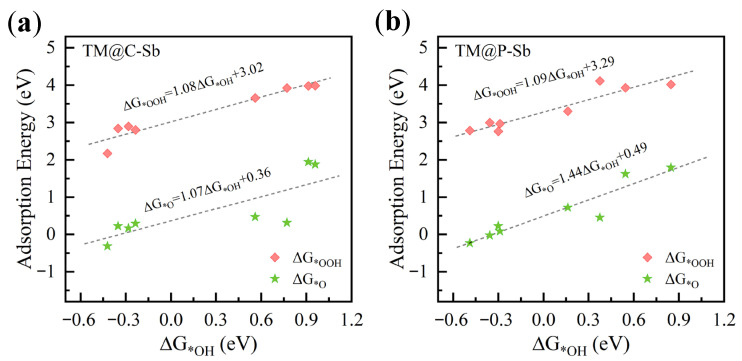
(**a**) TM@C–Sb and (**b**) TM@P–Sb are scaled relationships between intermediate adsorption energies, with the pink squares representing $\Delta G_{*OOH}$ vs. $\Delta G_{*OH}$ and the green pentagrams representing $\Delta G_{*O}$ vs. $\Delta G_{*OH}$.

In previous studies, strong linear scaling relationships have provided valuable insights into the surface characteristics of catalysts and catalytic mechanisms, helping to design catalysts more precisely for the optimization of specific reactions. The Gibbs free energies of three intermediates are considered potential descriptors for the binding strength between the catalyst’s active atoms and the intermediates. When constructing the free energy staircase for the TM@C/P–Sb system, we found a linear scaling relationship between ∆G*OH, ∆G*O, and ∆G*OOH. As shown in Figure 3a,b, there is a strong linear relationship between ∆G*OH and ∆G*OOH for the TM@C/P–Sb system. The regression equation for TM@C–Sb is ∆G*OOH = 1.08∆G*OH + 3.02, with a coefficient of determination (R^2^) of 0.93, which aligns well with other ORR catalysts; for TM@P–Sb, the regression equation is ∆G*OOH = 1.09∆G*OH + 3.29, with an R^2^ of 0.88. However, the linear equation between ∆G*O and ∆G*OH does not fit all the data, with R^2^ values of 0.63 and 0.89, respectively.

According to the Sabatier principle, the interaction strength between the adsorbate species and the catalytically active sites needs to be moderate. If the interaction is too strong, adsorbates like free radicals can bind too tightly to the active sites, hindering the adsorption and dissociation of subsequent reactants, thus leading to a decrease in catalytic activity. Conversely, if the interaction is too weak, the adsorbates cannot bind stably, making it difficult to effectively activate reactants and intermediates, which impedes the catalytic reaction. Therefore, a moderate interaction strength is crucial for achieving high catalytic activity, as it facilitates efficient reaction progression. The computational results of this study provide direct validation for this: the Cu@P–Sb system has the strongest binding with the OOH radical, and its ORR overpotential is higher (ηORR = 1.15 V), while the Cr@C–Sb system, with the lowest ΔG*OOH, has the weakest interaction, resulting in a higher ORR overpotential (ηORR = 1.65 V). This result indicates a clear correlation between the interaction strength and catalytic activity.

**Figure 4:**
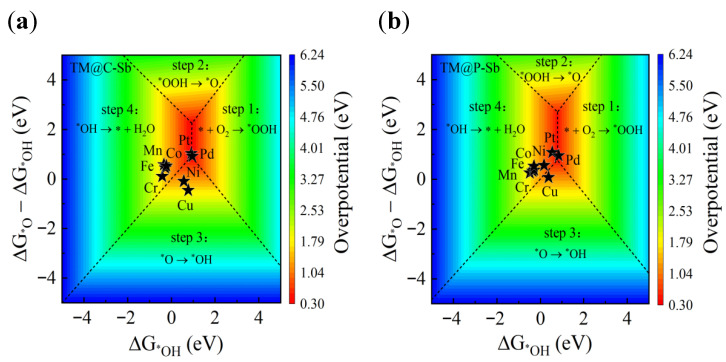
(**a**,**b**) The contour maps of the free energy changes during the ORR process for the TM@C–Sb and TM@P–Sb systems, respectively, where ηORR is expressed as a function of ΔGOH (x-axis) and ΔGO − ΔG*OH (y-axis). The contour lines divide the map into four regions, each corresponding to the free energy change of a different elementary step. The black stars in the figures represent different TM@C–Sb and TM@P–Sb.

When evaluating the electrocatalytic activity for the oxygen reduction reaction (ORR) on the TM@C/P–Sb materials, the change in ΔG is the key theoretical basis, with the descriptors ΔGOH and ΔGO − ΔG*OH forming the foundation for analysis. The activity contour maps (Figure 4a,b) created based on this can be divided into four distinct regions, each corresponding to an elementary step in the ORR and indicating that step as the rate-determining step (i.e., the rate-limiting step) for the entire reaction. The dashed lines between the regions in the contour map represent equal changes in free energy between adjacent steps, and catalysts located near the boundary between step 1 (* + O_2_ → OOH) and step 4 (OH → * + H_2_O) theoretically exhibit optimal ORR activity.

Analysis shows that for most TM@C/P–Sb systems, the adsorption of the OH intermediate is too strong, making step 4 (OH desorption to form water) the rate-determining step. In contrast, for the Cu@C–Sb, Cu@P–Sb, and Ni@C–Sb systems, the adsorption of OH is too weak, which shifts step 3 (O → *OH) to become the rate-determining step. In terms of overpotential, the calculated values for Pt@C–Sb (ηORR = 0.32 V), Pd@C–Sb (ηORR = 0.31 V), and Pd@P–Sb (ηORR = 0.38 V) are relatively low, demonstrating their potential as efficient ORR catalysts. The calculated overpotentials are lower than those reported for many previously studied ORR catalysts, indicating the enhanced catalytic performance of the proposed TM–NM co-doped systems. To further evaluate the catalytic performance, a comparison with previously reported ORR catalysts is summarized in SI Table S7. It can be seen that the TM–NM co-doped antimonene systems exhibit significantly lower overpotentials compared to single-atom catalysts and other reported systems.

In particular, Pd@C–Sb and Pt@C–Sb show superior performance, highlighting the effectiveness of the dual-doping strategy in optimizing the adsorption of reaction intermediates, as shown in SI Table S7.

In addition, the reaction energetics were compared with previously reported ORR systems. It is found that the free energy changes of the potential-determining steps in the TM–NM co-doped antimonene systems are smaller than those of typical single-atom catalysts and Fe–N–C systems reported in the literature [64]. This indicates that the energy barriers for the key reaction steps are effectively reduced, which is consistent with the observed low overpotentials and enhanced catalytic activity. This trend is consistent with the scaling relationship analysis discussed above.

### 3.4. Origin of Activity and Synergistic Mechanism Analysis

To gain deeper insight into the origin of the enhanced ORR performance in TM@C/P–Sb systems, the electronic structures, charge redistribution behavior, and bonding characteristics were systematically investigated.

First, the Bader charge analysis was conducted to quantify the charge transfer between the transition metal (TM) atoms and the neighboring nonmetal dopants. As summarized in SI, Table S5, both C and P atoms act as electron donors, transferring electrons to the TM centers. The magnitude of charge transfer depends strongly on the dopant species. Specifically, C atoms donate approximately 0.81–1.12 e to the TM atoms, whereas P atoms transfer a smaller amount of 0.32–0.75 e. This difference originates from the higher electronegativity of C (2.55) compared to P (2.19), which enables stronger electronic interaction between C and the TM atoms. As a result, electron-rich TM active centers are formed, which is beneficial for catalytic reactions involving oxygen-containing intermediates.

To further understand the bonding nature between the TM atoms and the dopants, crystal orbital Hamilton population (COHP) analysis was performed. The integrated COHP (ICOHP) values shown in SI Table S6 indicate that the TM–C bonds exhibit stronger bonding interactions (−2.31 to −3.36 eV) than TM–P bonds (−2.21 to −3.09 eV). The stronger TM–C bonding suggests enhanced orbital hybridization between the TM d orbitals and the C p orbitals. Such strong hybridization facilitates efficient charge redistribution between the TM center and surrounding atoms, which significantly modifies the electronic structure of the active site.

**Figure 5:**
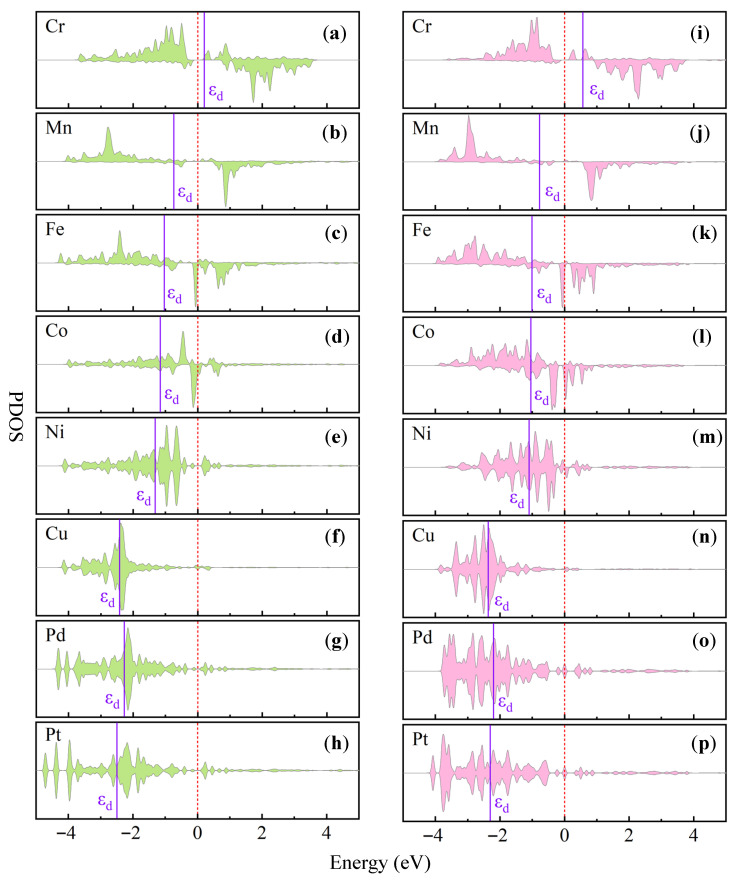
The projected density of states (PDOS) of the d-orbitals for the TM@C/P–Sb system is shown in the figure, where the position of the d-band center (εd) is marked in purple. The labels for the 3d transition metals from Cr to Cu are marked as (**a**–**f**,**i**–**n**); the 4d transition metal Pd is labeled as (**g**,**o**); and the 5d transition metal Pt is labeled as (**h**,**p**). The dashed line in the figure represents the Fermi level, with its energy set to 0 eV.

The electron donation from the nonmetal dopants, together with the strong TM–C/P orbital hybridization, directly affects the occupation of the TM d orbitals. As the d-electron occupation increases, the center of the d states gradually shifts away from the Fermi level. This behavior is reflected in the projected density of states (PDOS) analysis, as shown in Figure 5. The calculated d-band center (ε~d~) values of the TM atoms in TM@C–Sb systems follow the order

Cr (0.21 eV) > Mn (−0.74 eV) > Fe (−1.04 eV) > Co (−1.16 eV) > Ni (−1.32 eV) > Cu (−2.85 eV),

which correlates well with the increasing number of d electrons in the TM atoms. A similar trend is also observed in the TM@P–Sb systems.

**Figure 6:**
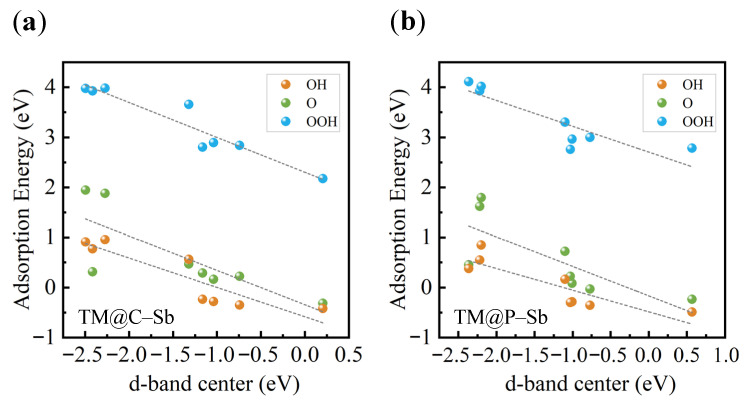
Relationship between the descriptor φ and the adsorption energies of *OH, *O, and *OOH for (**a**) TM@C–Sb and (**b**) TM@P–Sb.

According to the d-band theory, the position of the d-band center plays a crucial role in determining the interaction strength between the catalyst surface and adsorbates. A higher d-band center generally leads to stronger adsorption of reaction intermediates, whereas a lower d-band center weakens the adsorption strength. As illustrated in Figure 6, the adsorption free energies of key ORR intermediates (*OH, *O, and *OOH) exhibit clear linear correlations with the ε~d~ values, demonstrating that the d-band center serves as an effective electronic descriptor for ORR activity.

**Figure 7:**
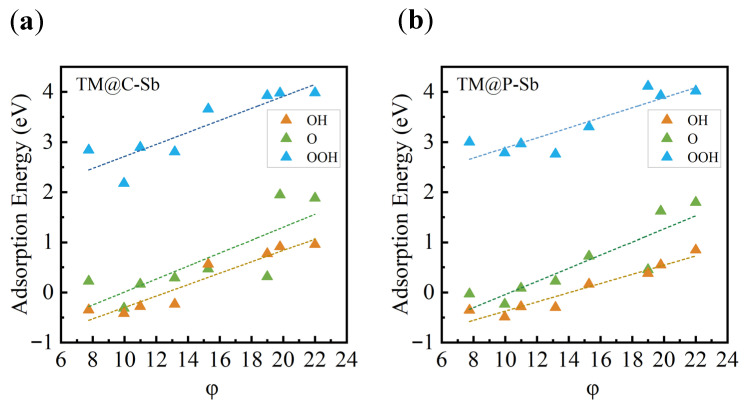
Linear correlation between the descriptor φ and the adsorption energies of *OH, *O, and *OOH for (**a**) TM@C–Sb and (**b**) TM@P–Sb.

In addition, a descriptor based on the intrinsic electronic characteristics of the TM atoms was introduced, defined as φ = N~e~ × χ, where N~e~ represents the number of d electrons and χ is the electronegativity of the TM atom. As shown in Figure 7, this descriptor also shows strong linear correlations with ΔG_OH_, ΔG_O_, and ΔG_*OOH_, further confirming that the intrinsic electronic configuration of the TM atoms governs the adsorption energetics of ORR intermediates.

Overall, the combined Bader charge and COHP analyses reveal that the nonmetal dopants synergistically regulate the electronic structure of TM centers through charge transfer and orbital hybridization. This process modifies the occupation of TM d orbitals and consequently shifts the d-band center, thereby optimizing the adsorption strength of oxygen-containing intermediates and ultimately enhancing the ORR catalytic activity of the TM@C/P–Sb systems.

Furthermore, this charge redistribution also provides insight into the origin of the scaling relationships observed in this work. The electron transfer between the TM atoms and the Sb substrate alters the electronic structure of the active sites, leading to a shift in the d-band center. As a result, the adsorption energies of key intermediates (*OOH, *O, and *OH) become correlated, giving rise to the scaling relationships commonly observed in ORR catalysis. This finding is consistent with previous studies, where charge transfer has been identified as a fundamental factor governing scaling relations [65].

To assess the stability of the optimized catalyst systems under practical operating conditions, ab initio molecular dynamics (AIMD) simulations were performed at 400 K for 8 ps on the Pd@C–Sb, Pt@C–Sb, Pd@P–Sb, and Pt@P–Sb systems, as shown in SI, Figure S2. The simulation results indicate that the total energy of each system fluctuates before stabilizing, with no significant structural deformation or atomic escape observed. These findings confirm that these catalysts possess excellent thermodynamic stability and structural integrity, highlighting their potential for practical applications.

## 4. Conclusions

Although the present study is purely theoretical, several computational indicators, including low vacancy formation energy of antimonene, strong dopant anchoring energies, and high thermal stability confirmed by AIMD, collectively suggest that these dual-atom configurations are experimentally feasible. Therefore, the results reported here can serve as practical guidance for the future experimental synthesis of high-performance antimonene-based ORR catalysts.

This work demonstrates that TM–NM co-doped antimonene is a promising candidate for ORR catalysis. In particular, Pd@C–Sb, Pt@C–Sb, and Pd@P–Sb exhibit outstanding ORR catalytic activity, delivering low overpotentials of 0.31 V, 0.32 V, and 0.38 V, respectively—markedly lower than their single-transition-metal-doped counterparts.

Mechanistic analyses reveal that nonmetal dopants (C and P) induce pronounced synergistic interactions with transition metal centers. Bader charge analysis indicates substantial electron transfer from the nonmetal atoms to the TM centers, forming electron-rich active sites, while COHP analysis confirms strong TM–C/P orbital hybridization that stabilizes the dual-atom configuration. These electronic interactions modify the occupation of TM d orbitals and consequently shift the d-band center, enabling effective regulation of the adsorption energetics of key ORR intermediates (*OOH, *O, and *OH) and thereby reducing the reaction overpotential.

Notably, the number of d-electrons of the transition metal is identified as a reliable electronic descriptor for evaluating intermediate binding strength and predicting ORR activity. In addition, scaling relationship analysis and activity contour mapping further elucidate the intrinsic activity trends and identify the rate-determining steps of the ORR process.

Ab initio molecular dynamics simulations confirm the excellent thermodynamic stability of the optimized catalysts. Overall, this study clarifies the atomic-scale origin of synergistic enhancement induced by dual-atom co-doping in antimonene, revealing a clear structure–electronic–activity relationship. The insights obtained here provide a rational design strategy for developing high-performance ORR electrocatalysts based on two-dimensional main-group materials. Future studies could extend the AIMD simulations to higher temperatures and longer time scales to further assess the thermal limits of these catalysts.

## Data Availability

The original contributions presented in this study are included in the article/supplementary material. Further inquiries can be directed to the corresponding authors.

## Supplementary Materials

The following supporting information can be downloaded at: https://www.mdpi.com/article/10.3390/nano16080465/s1, Note S1: Computational methods; Table S1: Binding energy Eb and cohesive energy Ec of TM@C/P–Sb systems (unit: eV); Table S2: The ΔG*OOH, ΔG*O and ΔG*OH values of the TM@C/P–Sb systems (all units in eV); Table S3: Calculated ORR elementary step free energies (ΔG1–ΔG4) and overpotentials (ηORR) for TM@C/P–Sb systems; Table S4: Adsorption free energies of Pd@C–Sb, Pt@C–Sb and Pd@P–Sb systems under GGA+U level (unit: V); Table S5: Bader charge analysis and d-band centers for TM@C/P–Sb systems; Table S6: ICOHP values for TM–C and TM–P bonds in TM@C/P–Sb systems; Table S7: Comparison of ORR overpotentials for different catalysts; Figure S1: Three-dimensional charge density difference maps for the Pd@C–Sb, Pt@C–Sb, Pd@P–Sb, and Pt@P–Sb systems; Figure S2: Total energy and temperature variations during AIMD simulations at 400 K for the Pd@C–Sb, Pt@C–Sb, Pd@P–Sb, and Pt@P–Sb systems.
