# Supplementary material for: Synergistic Regulation of Oxygen Reduction Activity on Antimonene via Transition Metal–Nonmetal Dual-Atom Doping
Source: Nanomaterials (Basel). 2026 Apr 14;16(8):465. doi: 10.3390/nano16080465 (PMC13119440; doi:10.3390/nano16080465)
Supplement: Supplementary file 1 [file nanomaterials-16-00465-s001.zip › nanomaterials-4229521-supplementary.pdf]

Synergistic Regulation of Oxygen Reduction Activity on Antimonene via Transition Metal–Nonmetal Dual-Atom Doping

Yusong Weng [1,2,†], Xin Zhao [1,2,†], Wentao Liang [1,2,†], Ming Wang [1,2], Wei Deng [3,4,]* and Xuefei Liu [1,2,]*

[1] School of Physics and Electronic Science, Guizhou Normal University, Guiyang 550025, China; 231108100030@gznu.edu.cn (Y.W.)
[2] School of Integrated Circuit, Guizhou Normal University, Guiyang 550025, China
[3] Guizhou Yiyun New Materials Technology Co., Ltd., Guiyang 561113, China
[4] School of Big Data Statistics, Guizhou University of Finance and Economics, Guiyang 550025, China
* Correspondence: wdeng@mail.gufe.edu.cn (W.D.); 201307129@gznu.edu.cn (X.L.)
† These authors contributed equally to this work.

Note S1.Computational methods

The binding energy (E_b) of TM and NM embedded in the defected Sb monolayer were calculated based on equation S1:

$$\mathrm{E_b = E_{TM@NM-Sb} - E_{V_{Sb}} - E_{TM} - E_{NM}} \quad \text{(S1)}$$

where $E_{TM@NM-Sb}$, $E_{V_{Sb}}$, E_{TM} and E_{NM} denote the total energy of the doped system, the energy with two neighboring Sb atom vacancies, TM and NM atoms, respectively. With such a definition, a more negative value of E_b indicates better thermodynamic stability.

The cohesive energies of bulk metal materials (E_c) can be obtained by equation S2:

$$\mathrm{E_c = (E_{bulk} - n \times E_{TM})/n} \quad \text{(S2)}$$

where the E_bulk is the energies of bulk metal, E_TM is the energies of single metal atoms, and n is the number of metal atom in its bulk structure.

The Gibbs free energy changes for the four elementary ORR steps from ΔG_1 to ΔG_4 can be calculated as S3-S6:

Academic Editor: Gregory M. Odegard

Received: 14 March 2026
Revised: 3 April 2026
Accepted: 9 April 2026
Published: 14 April 2026

Copyright: © 2026 by the authors. Licensee MDPI, Basel, Switzerland. This article is an open access article distributed under the terms and conditions of the Creative Commons Attribution (CC BY) license.

$$\Delta G_1 = G_{O_2} - G_{*O_2} \quad (S3)$$

$$\Delta G_2 = G_{*O_2} - G_{*OOH} + G(H^+ + e^-) \quad (S4)$$

$$\Delta G_3 = G_{*OOH} - G_{*OH} + G(H^+ + e^-) \quad (S5)$$

$$\Delta G_4 = G_{*OH} - G_{OH} \quad (S6)$$

Where G_{O_2} is the free energy of a system containing oxygen molecules, and G_{*O_2}, G_{*OOH}, G_{*OH}, and G_{OH} represent the free energy of a system containing the corresponding intermediates, respectively. H^+ and e^- represent the free energies of protons and electrons.

Due to the poor description of the high-spin ground state of O_2 by DFT, it is necessary to use $G(O_{2,}g) + 2G(H_{2,}g) - 2G(H_2O, l) = 4.92$ eV to estimate its free energy. In general, the Gibbs free energy of liquid-phase H_2O is difficult to calculate directly, but at equilibrium conditions, the difference between the Gibbs free energies of the liquid and the gas is zero. This allows us to use the gas-phase H_2O data to estimate the liquid-phase properties, which can be calculated from S7:

$$G(H_2O, l) = E_{H_2O} + ZPE_{H_2O}\text{-}TS_{H_2O} \quad (S7)$$

where E_{H_2O}, ZPE_{H_2O} and TS_{H_2O} are the total energy of H_2O from DFT calculations in gas phase, zero-point energy and entropy respectively.

The changes of free energy in ORR can be described as:

$$\Delta G_1 = G_{*OOH} - 4.92 \quad (S8)$$

$$\Delta G_2 = G_{*O} - G_{*OOH} \quad (S9)$$

$$\Delta G_3 = G_{*OH} - G_{*O} \quad (S10)$$

$$\Delta G_4 = -G_{*OH} \quad (S11)$$

The overpotential of ORR can be found from the following equation S12:

$$\eta_{ORR} = \max(\Delta G_1, \Delta G_2, \Delta G_3, \Delta G_4)/e + 1.23 \quad (S12)$$

where 1.23 is the equilibrium potential introduced in the free energy change.

Table. S1 The binding energy E_b and cohesive energy E_c of TM@C/P-Sb systems with unit eV.

TM	TM@C-Sb		TM@P-Sb	
	E_b	E_c	E_b	E_c
Cr	-10.48	-5.08	-8.62	-5.08
Mn	-9.38	-3.79	-7.49	-3.79
Fe	-9.86	-4.96	-7.98	-4.96
Co	-10.29	-5.28	-8.43	-5.28
Ni	-10.23	-4.86	-8.40	-4.86
Cu	-8.62	-3.49	-7.17	-3.49
Pd	-9.80	-3.73	-8.30	-3.73
Pt	-11.98	-5.56	-10.19	-5.56

Table. S2 The ΔG_{*OOH}, ΔG_{*O} and ΔG_{*OH} values of the TM@C/P-Sb systems (all units in eV).

TM	TM@C-Sb			TM@P-Sb		
	ΔG_{*OOH}	ΔG_{*O}	ΔG_{*OH}	ΔG_{*OOH}	ΔG_{*O}	ΔG_{*OH}
Cr	2.17	-0.32	-0.42	2.78	-0.23	-0.49
Mn	2.84	0.22	-0.35	3.00	-0.03	-0.36
Fe	2.89	0.17	-0.28	2.96	0.08	-0.29
Co	2.80	0.29	-0.24	2.76	0.22	-0.30
Ni	3.66	0.47	0.56	3.30	0.72	0.16
Cu	3.92	0.31	0.77	4.11	0.45	0.38
Pd	3.98	1.88	0.96	4.02	1.79	0.85
Pt	3.98	1.94	0.91	3.93	1.62	0.55

Table. S3 The calculated each elementary step ΔG_1, ΔG_2, $\Delta G_3$and ΔG_4 for ORR (all units in eV); The overpotential of ORR (η) for TM@C/P-Sb (all units in V).

TM@C-Sb	ΔG_1 (eV)	ΔG_2 (eV)	ΔG_3 (eV)	ΔG_4 (eV)	η_{ORR} (V)
Cr	0.91	1.03	2.03	0.94	1.65
Mn	0.96	0.92	2.10	0.94	1.58
Fe	-0.42	0.10	2.49	2.75	1.51
Co	-0.35	0.58	2.61	2.08	1.47
Ni	-0.28	0.45	2.73	2.03	1.32
Cu	-0.24	0.52	2.51	2.12	1.69
Pd	0.56	-0.09	3.19	1.26	0.31
Pt	0.77	-0.46	3.61	1.00	0.32
TM@P-Sb	ΔG_1 (eV)	ΔG_2 (eV)	ΔG_3 (eV)	ΔG_4 (eV)	η_{ORR} (V)
Cr	-0.49	0.26	3.02	2.14	1.72
Mn	-0.36	0.33	3.03	1.92	1.59
Fe	-0.29	0.37	2.88	1.96	1.52
Co	-0.30	0.52	2.54	2.16	1.53
Ni	0.16	0.56	2.58	1.62	1.07
Cu	0.38	0.08	3.66	0.81	1.15
Pd	0.85	0.95	2.22	0.90	0.38
Pt	0.55	1.08	2.31	0.99	0.68

Table. S4 The calculated adsorption free energies of Pd@C-Sb, Pt@C-Sb and Pd@P-Sb systems under GGA+U level (all units in V).

<table>
<tr><td rowspan="6">η_{ORR}</td><td rowspan="2">Pd@C-Sb</td><td>U = 0</td><td>0.3112</td></tr>
<tr><td>U = 3.3</td><td>0.3799</td></tr>
<tr><td rowspan="2">Pt@C-Sb</td><td>U = 0</td><td>0.3167</td></tr>
<tr><td>U = 2.4</td><td>0.3139</td></tr>
<tr><td rowspan="2">Pd@P-Sb</td><td>U = 0</td><td>0.3833</td></tr>
<tr><td>U = 3.3</td><td>0.4004</td></tr>
</table>

Table. S5 Bader charge analysis and d-band centers for TM@C/P-Sb systems.

System	Atom	Original charge (e)	Final charge (e)	Charge transfer (e)	εd (eV)	d-occupation
TM@C-Sb						
Pd@C-Sb	C	4	4.89	+0.89	–2.272	9.25
	Pd	10	10.19	+0.19		
Pt@C-Sb	C	4	4.81	+0.81	–2.495	6.30
	Pt	10	10.39	+0.39		
Co@C-Sb	C	4	4.92	+0.92	–1.163	7.69
	Co	9	8.85	–0.15		
Cr@C-Sb	C	4	5.09	+1.09	+0.206	8.28
	Cr	12	10.90	–1.10		
Cu@C-Sb	C	4	5.00	+1.00	–2.416	7.75
	Cu	11	10.82	–0.18		
Fe@C-Sb	C	4	5.04	+1.04	–1.037	8.10
	Fe	8	7.49	–0.51		
Mn@C-Sb	C	4	5.12	+1.12	–0.741	8.58
	Mn	13	12.19	–0.81		
Ni@C-Sb	C	4	4.92	+0.92	–1.320	7.11
	Ni	10	9.95	–0.05		
TM@P-Sb						
Pd@P-Sb	P	5	5.42	+0.42	–2.198	7.55
	Pd	10	10.31	+0.31		
Pt@P-Sb	P	5	5.32	+0.32	–2.220	6.7
	Pt	10	10.61	+0.61		
Co@P-Sb	P	5	5.57	+0.57	–1.031	6.49
	Co	9	8.81	–0.19		
Cr@P-Sb	P	5	5.70	+0.70	+0.567	7.19
	Cr	12	10.92	–1.08		
Cu@P-Sb	P	5	5.61	+0.61	–2.364	8.77
	Cu	11	10.94	–0.06		
Fe@P-Sb	P	5	5.67	+0.65	–1.008	7.96

	Fe	8	7.55	–0.45		
Mn@P-Sb	P	5	5.75	+0.75	–0.772	9.67
	Mn	13	12.18	–0.82		
Ni@P-Sb	P	5	5.51	+0.51	–1.100	8.08
	Ni	10	10.05	+0.05		

Table. S6 ICOHP values for TM–C and TM–P bonds in TM@C/P-Sb systems.

System	ICOHP (eV)	System	ICOHP (eV)
Pd@C-Sb	–2.57	Pd@P-Sb	–2.51
Pt@C-Sb	–3.36	Pt@P-Sb	–3.09
Co@C-Sb	–3.16	Co@P-Sb	–2.85
Cr@C-Sb	–2.89	Cr@P-Sb	–2.21
Cu@C-Sb	–2.31	Cu@P-Sb	–2.55
Fe@C-Sb	–3.06	Fe@P-Sb	–2.85
Mn@C-Sb	–2.55	Mn@P-Sb	–2.28
Ni@C-Sb	–2.84	Ni@P-Sb	–2.74

Table S7 Comparison of ORR overpotentials for different catalysts.

Catalyst	Substrate	Overpotential (V)	Reference
Pd@C–Sb	Antimonene	0.31	This work
Pt@C–Sb	Antimonene	0.32	This work
Pd@P–Sb	Antimonene	0.38	This work
Pd@Sb	Antimonene	0.58	[62]
Pt@Sb	Antimonene	0.71	[63]
Fe–N–C	Graphene	~0.45	[11]

Figure S1 (a) ~ (d) show the top and side views of the three-dimensional charge density difference maps for the Pd@C-Sb, Pt@C-Sb, Pd@P-Sb, and Pt@P-Sb systems, respectively; the charge density value is 0.003 $e/Å^3$; the electron depletion and accumulation regions are represented on the light blue and yellow isosurfaces, respectively.

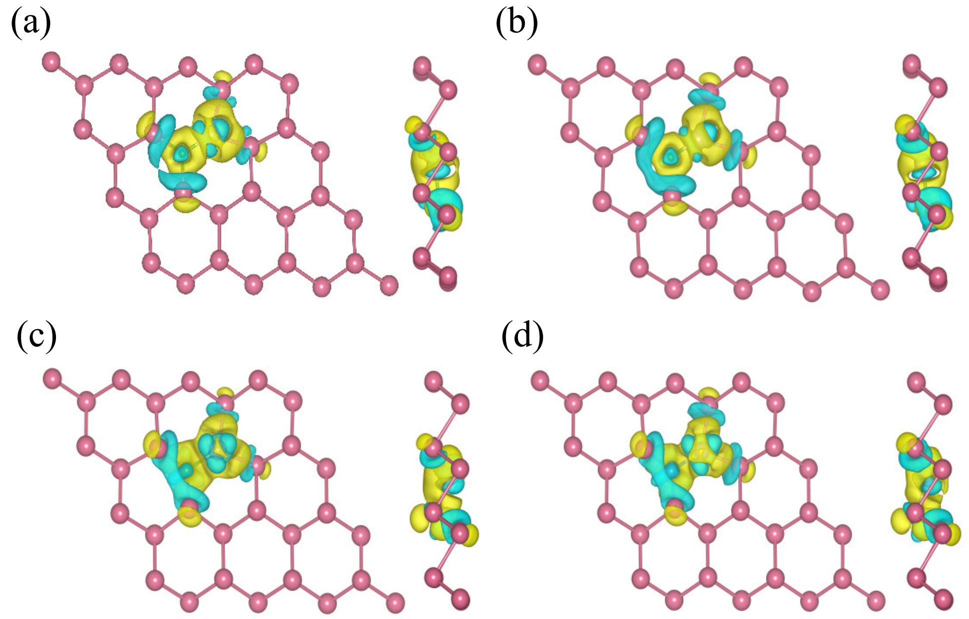

Figure S2 (a) ~ (d) respectively show the variations in total energy and temperature at the target temperature of 400 K during AIMD simulations for the Pd@C-Sb, Pt@C-Sb, Pd@P-Sb, and Pt@P-Sb systems. The insets on the left and right sides of each figure illustrate the structural schematics of the systems before and after the AIMD process.

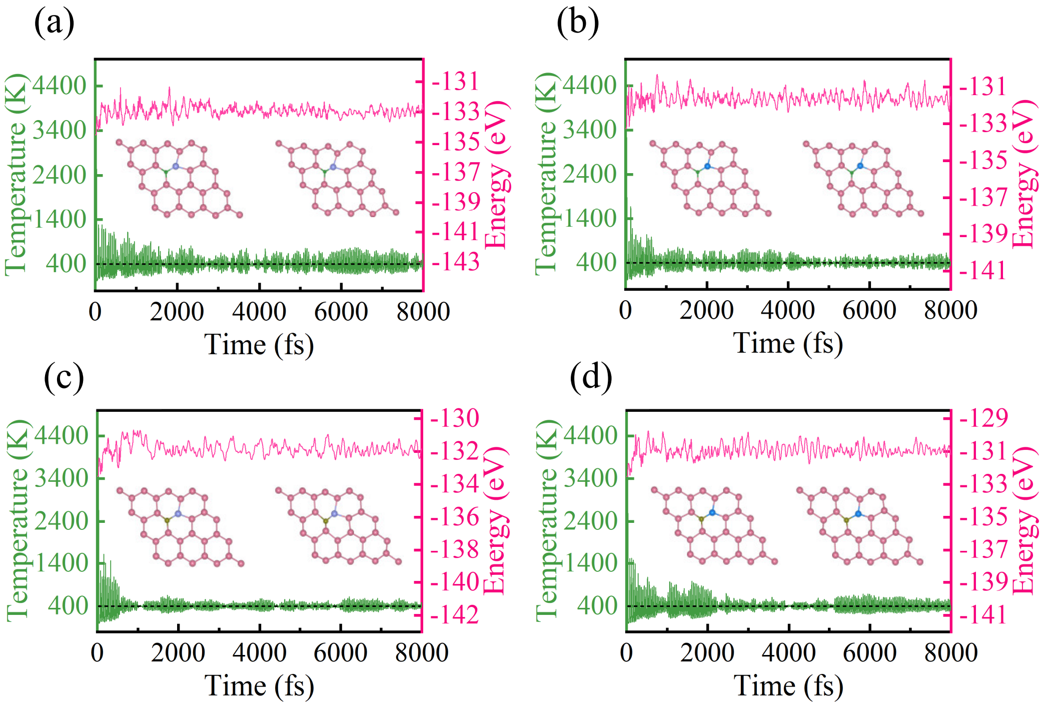

(a)
(b)
(c)
(d)
Temperature (K)
Energy (eV)
Time (fs)
